# Decoupled MOF Breathing: Pressure‐Induced Reversal of Correlation Between Orthogonal Motions in a Diamondoid Framework

**DOI:** 10.1002/anie.202504297

**Published:** 2025-05-15

**Authors:** David J. Ashworth, Elliot J. Carrington, Thomas M. Roseveare, Charles J. McMonagle, Martin R. Ward, Ashleigh J. Fletcher, Tina Düren, Mark R. Warren, Stephen A. Moggach, Iain D. H. Oswald, Lee Brammer

**Affiliations:** ^1^ Department of Chemical and Process Engineering University of Strathclyde Montrose Street Glasgow G14 1XJ UK; ^2^ Strathclyde Institute of Pharmacy & Biomedical Sciences (SIPBS) University of Stracthclyde 161 Cathedral Street Glasgow G4 0RE UK; ^3^ Department of Chemistry University of Sheffield Brook Hill Sheffield S3 7HF UK; ^4^ European Synchrotron Research Facility 71 Avenue des Martyrs Grenoble 38000 France; ^5^ Centre for Integrated Materials Processes and Structures and Department of Chemical Engineering University of Bath Claverton Down Bath BA2 7AY UK; ^6^ Diamond Light Source Harwell Science and Innovation Campus Didcot OX11 0DE UK; ^7^ Centre for Microscopy Characterisation and Analysis and School of Molecular Science The University of Western Australia 35 Stirling Highway, Crawley Perth 6009 Australia

**Keywords:** Breathing, High pressure, In‐situ diffraction, Metal‐organic framework, Switching

## Abstract

Responsive porous materials can outperform more rigid analogues in applications requiring precise triggering of molecular uptake/release, switching or gradual change in properties. We have uncovered an unprecedented dynamic response in the diamondoid MOF **SHF‐62**, (Me_2_NH_2_)[In(BDC‐NHC(O)Me)_2_] (BDC = 1,4‐benzenedicarboxylate), by using pressure as a stimulus. **SHF‐62** exhibits two distinct framework “breathing” motions involving changes in 1) cross‐section and 2) length of its 1D pores. Our study using synchrotron single‐crystal X‐ray diffraction in sapphire‐capillary (*p* < 0.15 GPa) and diamond‐anvil (0.15 < *p* < 5 GPa) cells reveals that different pressure regimes trigger positive and negative correlation between these two motions, requiring an unprecedented mechanical decoupling. Specifically, the DMF‐solvated framework **SHF‐62‐DMF**, in DMF as pressure‐transmitting medium, undergoes initial hyperexpansion of pore cross‐section (*p* ≤ 0.9 GPa), due to DMF ingress, followed by reversal/reduction at *p* > 0.9 GPa while pore length contracts for *all* pressure increases, revealing *decoupling* of the two framework deformations. By contrast, nonpenetrating medium FC‐70 imposes correlated compression (*p* < 1.4 GPa) of pore cross‐section and length, resembling framework activation/desolvation motions but of greater magnitude. Similar behavior occurs for **SHF‐62‐CHCl_3_** in CHCl_3_ (*p* < 0.14 GPa), suggesting minimal ingress of CHCl_3_. These findings change our understanding of MOF dynamic responses and provide a platform for future responsive materials development.

## Graphical abstract

The diamondoid MOF SHF‐62 exhibits “breathing” motions affecting the cross‐section and length of its 1D pores. Application of pressure reveals that the two motions can be switched between positive correlation and negative correlation, demonstrating decoupling of these motions within the 3D framework and providing a platform for design of more complex dynamic responsive materials.

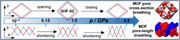

## Introduction

Metal‐organic frameworks (MOFs) have established a prominent role in porous materials research over the past 25+ years. Their modular construction enables chemical and spatial tunability and affords myriad applications that primarily exploit the internal surface area of their pores.^[^ ^1^, ^2^, ^3^ ^]^ Only a small proportion of MOFs are reported as flexible materials, but these are being investigated in increasing numbers in relation to their dynamic deformation in response to external stimuli such as temperature, pressure, light, and adsorbed species content.^[^ ^4^, ^5^ ^]^ Such responsive materials offer the potential to outperform more rigid porous materials in applications where precise triggering of uptake or release of guest molecules, switching behavior or gradual change in properties is of importance, including selective separations,^[^ ^6^ ^]^ catalysis,^[^ ^7^ ^]^ and targeted release.^[^ ^8^ ^]^ Structure–property relationships for flexible framework materials remain underexplored, however, and their mapping is essential to understand the fundamental molecular‐level dynamics that enable structural flexibility.^[^ ^9^ ^]^

Pressure is one of the most recent extensions of stimulus‐response investigations in MOFs and is a potent tool to explore their flexibility.^[^ ^10^, ^11^, ^12^, ^13^ ^]^ Understanding the effects of the application of pressure is important for these materials, as it can interrogate diverse mechanical properties including compressibility and stability, as well as enabling exploration of phase diversity.^[^ ^11^ ^]^ In addition, changes in structure on compression may have important repercussions for applied properties such as adsorption selectivity and specificity,^[^ ^14^, ^15^ ^]^ transport properties,^[^ ^16^, ^17^ ^]^ electronics,^[^ ^18^, ^19^ ^]^ and pressure‐driven sensors.^[^ ^20^, ^21^ ^]^

Given that processing and operating conditions for MOFs usually deviate from controlled room temperature and pressure laboratory conditions (1 atm, 20 °C),^[^ ^22^ ^]^ it is essential to understand the landscape of the dynamic behavior of MOFs in response to pressure. Pressure is an important component of building the experimental and conceptual platform that underpins the development of new materials tailored toward specific applications. Such understanding requires detailed structural information. Thus, single‐crystal X‐ray diffraction (SCXRD) studies (0.1 GPa < *p* < 10 GPa; 1 GPa = 10 000 bar) using diamond‐anvil cells (DACs)^[^ ^23^ ^]^ have been used to identify different structural behaviors under pressure for commonly studied MOFs, sometimes in conjunction with supporting computational modelling techniques.^[^ ^24^, ^25^, ^26^, ^27^ ^]^ Such behaviors, induced in MOFs under pressure, include pore‐volume “breathing,”^[^ ^28^, ^29^, ^30^ ^]^ linker rotations that facilitate pore access,^[^ ^31^, ^32^ ^]^ polymorphic phase transitions,^[^ ^33^, ^34^ ^]^ negative linear compression,^[^ ^29^, ^35^, ^36^ ^]^ and reversible/irreversible amorphization.^[^ ^37^, ^38^, ^39^ ^]^ Pressure‐induced alterations of material properties can include luminescence,^[^ ^40^, ^41^, ^42^ ^]^ stability,^[^ ^28^, ^37^ ^]^ mechanical properties^[^ ^43^ ^]^ and guest uptake.^[^ ^32^ ^]^ However, some behaviors may occur at pressures too low to be accessed and mapped via traditional DACs^[^ ^44^, ^45^, ^46^ ^]^ of the Merrill–Bassett design^[^ ^23^, ^47^ ^]^ due to the minimum pressure and steps that can be applied using these cells (*p*_min_ ≈ 0.1–0.15 GPa and pressure steps Δ*p* > 0.1 GPa).

The recent development of a sapphire capillary cell (SCC) at beamline I19 at Diamond Light Source,^[^ ^48^ ^]^ provides the opportunity to investigate materials under lower pressure conditions and with precise application of pressure.^[^ ^49^ ^]^ The SCC comprises a mechanically robust and optically transparent chamber that enables rapid in situ control of the mid‐pressure regime, covering 20–1500 bar, with 1 bar precision. This pressure range is particularly pertinent to the study of MOFs as they become integrated into industrial applications. The effect of materials processing (shaping and tabletting, for example) will expose these materials to pressure changes in this range.^[^ ^50^, ^51^ ^]^ The SCC can be used in tandem with DAC studies to cover a wider high‐pressure regime with improved precision of pressure measurement and access to a greater proportion of reciprocal space in diffraction experiments.^[^ ^52^ ^]^

We have previously demonstrated that the twofold interpenetrated, diamondoid MOF **SHF‐61**, (Me_2_NH_2_)[In(BDC‐NH_2_)_2_] (BDC‐NH_2_ = amino‐1,4‐benzenedicarboxylate), exhibits continuous large‐amplitude, predominantly two‐dimensional (2D: *b*‐ and *c*‐axes) “breathing” behavior in response to removal of guest molecules.^[^ ^53^ ^]^ The flexible range accessible to the framework is highly dependent on the guest species present within the pore channels. From the CHCl_3_‐filled framework, the MOF can be activated, by removal of CHCl_3_, to leave a highly porous solid enabling a large uptake of CO_2_. By contrast, activation of the DMF‐filled framework results in a large magnitude structural change (−Δ*V*_unit cell_ > 15%), whereby removal of DMF leads to closing of the pore channels, which has been crystallographically mapped along a continuum of structural evolution. This activated form, after removal of DMF, exhibits limited gas adsorption (CO_2_, CH_4_, and N_2_), whereas a partially solvated state can be refilled with CO_2_ to a fully open state. This unusual behavior indicated intriguing pore‐solvent‐mediated interframework interactions of the interpenetrated networks.

In a bid to further develop responsive materials, we synthesized **SHF‐62**,^[^ ^54^ ^]^ a postsynthetically modified derivative of **SHF‐61**, with pendant methylamide groups replacing the amine functionality while retaining the symmetry of the parent material (space group *Fddd*).^[^ ^53^ ^]^ This modification results in a reduction in amplitude of the pore cross‐section deformation upon solvent removal, but a large increase in the pore‐length motion (*a*‐axis), which leads to a distinctly 3D breathing motion in **SHF‐62**.^[^ ^54^ ^]^ These motions can be mapped crystallographically to show dynamic 2D pore closing behavior (characterized by the *b*‐ and *c*‐axes), accompanied by significant 1D contraction of the framework helices parallel to the pore channel (*a*‐axis). Throughout activation studies, the direction of these motions is positively correlated, i.e., pore closure is accompanied by pore length contraction.^[^ ^55^ ^]^ In a significant deviation from the parent **SHF‐61** behavior, **SHF‐62** exhibits a similar structural response to the removal of either CHCl_3_ or DMF as pore solvent, i.e., solvent‐dependent behavior is lost.

**Scheme 1:**
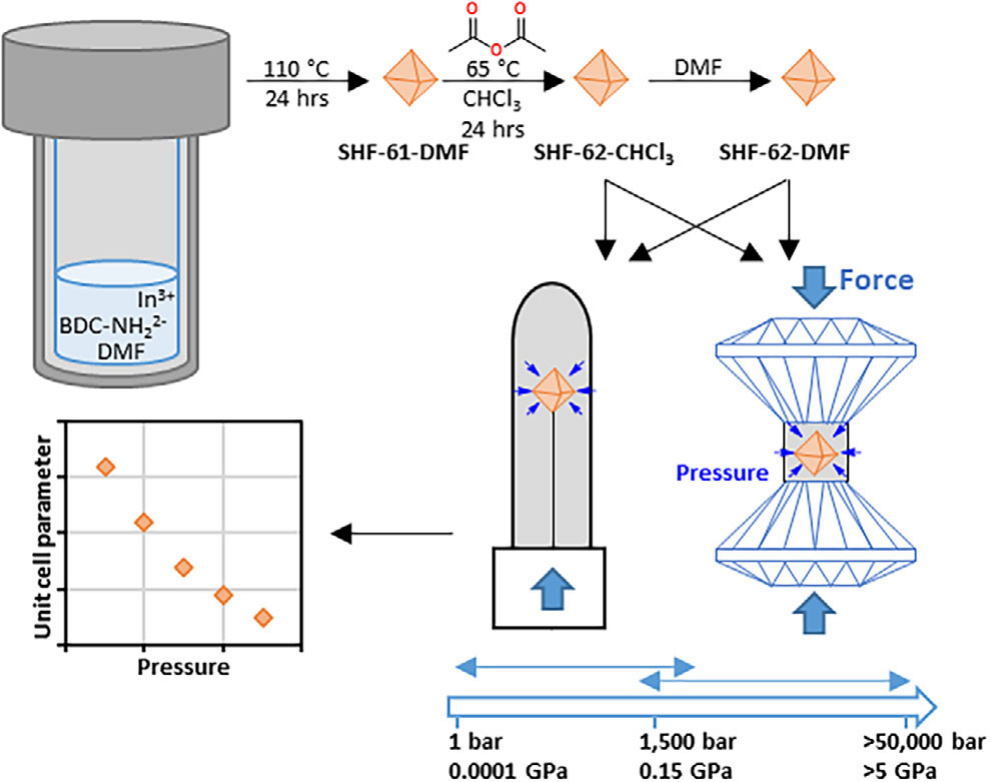
Overview of synthesis methodology, pressure studies, and analyses.

In this work, we map the structural response of the framework **SHF‐62** to a different stimulus: pressure. We employ a combination of pressure cells (SCC and DAC) to provide a detailed picture of the structural response to pressure, generated using different types of pressure‐transmitting media (PTMs), across multiple pressure regimes (Scheme 1). This work demonstrates the potential of this tandem approach to understand nuances of framework flexibility. In particular, the study reveals the different responses to pressure in different media, the previously unknown pressure‐driven hyperexpansion and compression of the MOF and, most remarkably, the pressure‐induced decoupling of the orthogonal framework motions that involve changes in pore cross‐section and in pore length, respectively. Revelation of this unprecedented mechanical behavior provides a platform for future development of applications that can harness such behavior, e.g. pressure sensing, and can act as a stimulus for further research to seek other triggers for such dynamic behavior in related materials that can open pathways to other applications. We also show that pressure has the potential to serve as a proxy stimulus for activation in MOFs to understand the structural deformations at the onset of framework collapse in other MOFs that are unstable under activation.

## Results and Discussion

**Figure 1:**
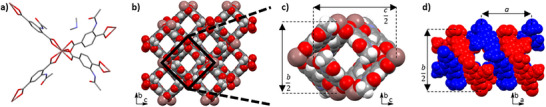
Crystal structure of **SHF‐62** (here as **SHF‐62‐DMF**) showing a) In(L)_4_ coordination environment of the metal centers (L = O_2_CC_6_H_3_(NC(=O)Me)CO_2_), with neighboring Me_2_NH_2_^+^ counterion, and b) view down *a*‐axis, showing lozenge‐shaped pores. Hydrogen atoms omitted for clarity in (a). c) View of one pore showing cross‐section dimensions. d) View down *c*‐axis with helical network arrangement propagating along pore length (*a*‐axis); the two interpenetrated networks are shown in red and blue.

**SHF‐62** (Figure 1a,b) was synthesized in its dimethylformamide (DMF) and chloroform (CHCl_3_) solvated forms (**SHF‐62‐DMF** and **SHF‐62‐CHCl_3_**, respectively) according to our previous methodology (Section S2 for details) involving solvothermal synthesis, postsynthetic modification, and pore‐solvent exchange,^[^ ^54^ ^]^ yielding single crystals that are stable for extended periods of time (months) when stored under the respective solvent. These solvated forms retain the doubly interpenetrated diamondoid network arrangement of the parent **SHF‐61** material in which lozenge‐shaped pores contain charge‐balancing Me_2_NH_2_^+^ cations and solvent molecules. Pore cross‐section is readily monitored by the correlated changes in crystallographic *b*‐ and *c*‐axes (Figure 1c), which can be used to define pore opening (*O*, %, see Section S3), and pore length is represented by the *a*‐axis (Figure 1d). The amide substituents are located exclusively at one of the four ring sites. Although this crystallographic ordering is unusual in MOFs comprising monosubstituted 1,4‐BDC linker ligands, here it is consistent with the prominent role of the substituent in linking the two interpenetrated networks via noncovalent interactions.^[^ ^53^, ^54^ ^]^

### Nonpenetrating Medium (FC‐70) – Impact of External Pressure

**Figure 2:**
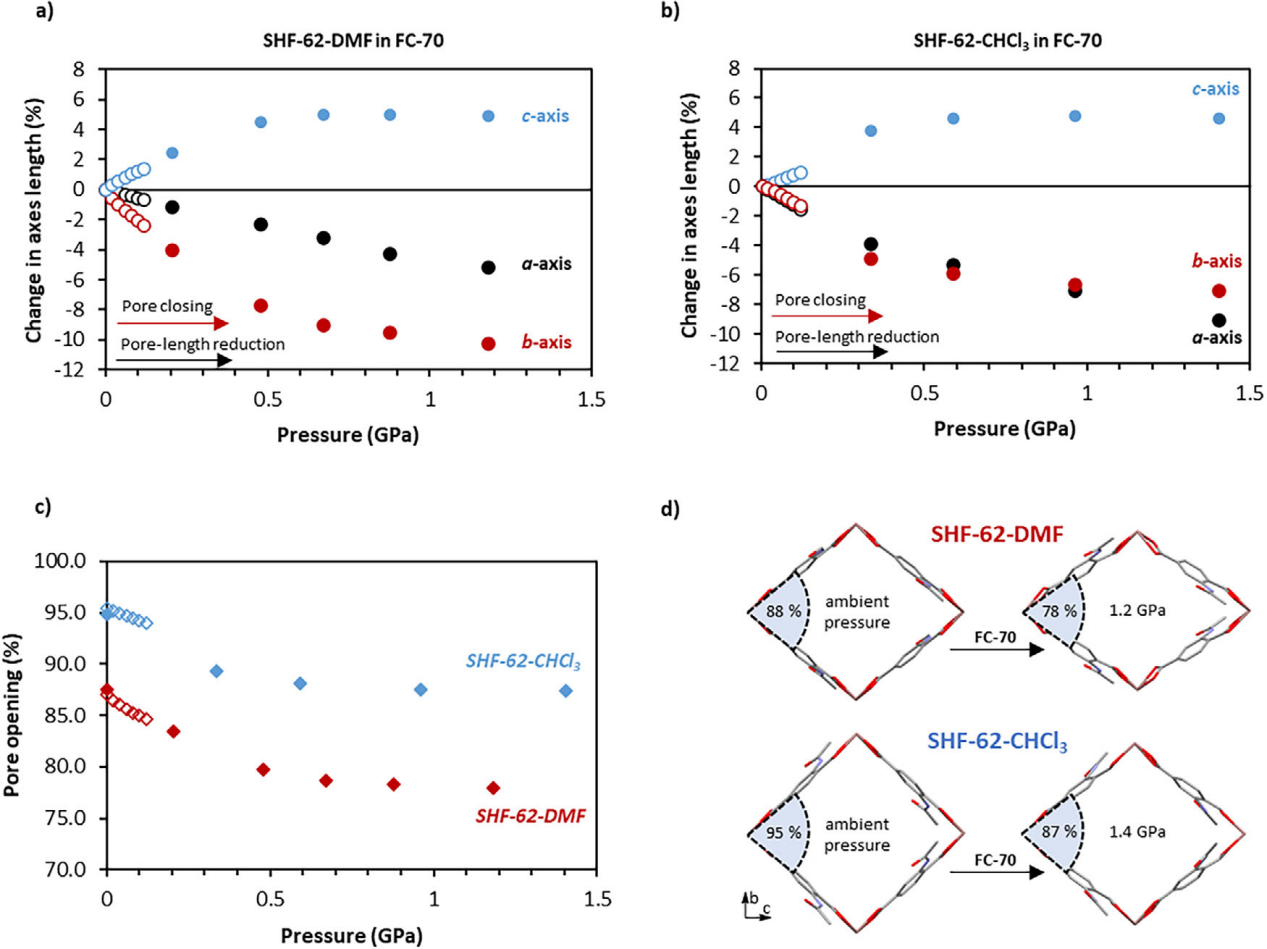
Changes to unit cell parameters for a) **SHF‐62‐DMF** and b) **SHF‐62‐CHCl_3_** with increasing pressure applied by nonpenetrating PTM FC‐70, expressed as change in unit cell axis lengths. c) Change of pore opening, *O*, as a function of pressure. SCC studies are represented by open symbols and DAC studies as filled symbols. d) Structural models of **SHF‐62‐DMF** and **SHF‐62‐CHCl_3_** at ambient pressure and at maximum pressure in FC‐70 with indication of pore opening, *O*. Pore opening (*O*, %) is calculated from a geometric projection of the framework onto a 2D surface; *O* (%) = tan^−1^(*b*/*c*) x (100/45°), see Section S3.

Single crystals of **SHF‐62‐CHCl_3_** and **SHF‐62‐DMF** were exposed to pressure applied using the trisperfluoroalkylamine FC‐70 as a nonpenetrating pressure‐transmitting medium (PTM). Studies in both a SCC and DAC enabled comparison of the MOF structural response across the two pressure regimes (SCC, *p* < 0.15 GPa; DAC, *p* > 0.15 GPa; Figure 2); for consistency throughout the manuscript, all pressures are hereafter expressed in GPa. In the SCC, pressure was initially increased to 0.002 GPa to dissolve any air trapped in the capillary during sample loading. X‐ray data collections were recorded at *p* = 0.002 GPa and at 0.02 GPa pressure intervals up to 0.12 GPa. For DAC studies, X‐ray data were collected at the initial loading pressure and several pressures in the range 0.2–1.5 GPa. The starting unit‐cell dimensions for **SHF‐62‐CHCl_3_** and **SHF‐62‐DMF** indicate that of the crystals selected, the former was more open (*O*_CHCl3_ = 95% and *O_DMF_* = 88%), which is consistent with previous observations.^[^ ^54^ ^]^ We ascribe this observation to differences in solvent‐framework interactions between solvation with CHCl_3_ versus DMF. The behavior on compression in FC‐70 across the accessible pressure range is similar for the two solvated forms. The *a*‐axis shortens, due to compression of the spring‐like framework helices (Figure 2a,b), in combination with a decrease in pore opening, *O* (from 95% to 87% for **SHF‐62‐CHCl_3_** and from 88% to 78% for **SHF‐62‐DMF**), due to a decrease in the *b*‐axis coupled with an increase in the *c*‐axis (Figure 2c). In the highest pressure (DAC) studies, measurements were limited to *p* ≤ 1.4 GPa due to a reduction in diffraction quality at higher pressures.

The decreases in overall unit cell volumes are similar in magnitude for **SHF‐62‐DMF** (Δ*V* = −10.7 %) and **SHF‐62‐CHCl_3_** (Δ*V =* −11.6 %). There are some differences in response along different directions, however, which we ascribe to the presence of different pore‐solvents. Thus, **SHF‐62‐DMF** shows the more restricted compression of the pore length (*a*‐axis), but an increased compressibility of the *b*‐axis (**SHF‐62‐DMF**: Δ*a* = −5.2%, Δ*b* = −10.2%, and Δ*c* = +4.9% versus **SHF‐62‐CHCl_3_**: Δ*a* = −9.1%, Δ*b* = −7.0%, and Δ*c* = +4.6%). Both materials show a good fit to a 3^rd^ order Birch–Murnaghan equation of state (Figure S10 and Table S6). The calculated bulk moduli of 5.6(4) and 4.9(9) GPa (Section S4.3)^[^ ^56^ ^]^ for **SHF‐62‐DMF** and **SHF‐62‐CHCl_3_**, respectively, lie toward the lower end of the range of bulk moduli reported for archetypal MIL‐53 breathing MOFs (3‐11 GPa),^[^ ^11^, ^57^, ^58^ ^]^ indicating that flexible “breathing” MOFs such as **SHF‐62** are sensitive to “small” perturbations of external pressure, as observed in the SCC experiments. In comparison, MOFs commonly described as rigid, such as HKUST‐1, MOF‐5, and UiO‐66, exhibit bulk moduli typically of 15–40 GPa.^[^ ^11^ ^]^ The SCC studies were also used to establish that the dynamic response was reversible on decompression (Figures S6 and S7). Reversibility was not explored at the higher pressures accessible in the DAC, but we note that, in other MOFs, reversible amorphization under pressure has been observed.^[^ ^37^, ^38^, ^59^ ^]^ This can result from localized deformations of the structure in response to high pressure, resulting in a loss of long‐range order, but when pressure is released the order is regained.

### Penetrating Medium (DMF) – Competition Between Internal and External Pressure

**SHF‐62‐DMF** was pressurized using DMF as the PTM in the SCC and DAC, with X‐ray diffraction studies conducted in an analogous manner to those using FC‐70. The SCC study (*p* < 0.14 GPa) revealed a new dynamic response, distinct from that observed under FC‐70. Upon pressure increase, the pore length still decreases (Δ*a* < 0), involving compression of the framework helices. This compression, however, is now accompanied by an *opening* of the pores (Δ*b* > 0; Δ*c* < 0), thereby converting the positive correlation between changes to pore length and pore cross‐section (previously observed with FC‐70) into a negative correlation. Stepwise reduction in pressure indicates the behavior is reversible (Supporting Information Figure S8), although the relaxed structure exhibits longer and more open pores than the starting crystal with a net increase in unit cell volume (Δ*V* ≈ 58.1(2) Å^3^, 0.5%). This behavior is consistent with ingress of DMF molecules into the MOF channels under pressure and some retention of additional DMF upon release of the pressure.

**Figure 3:**
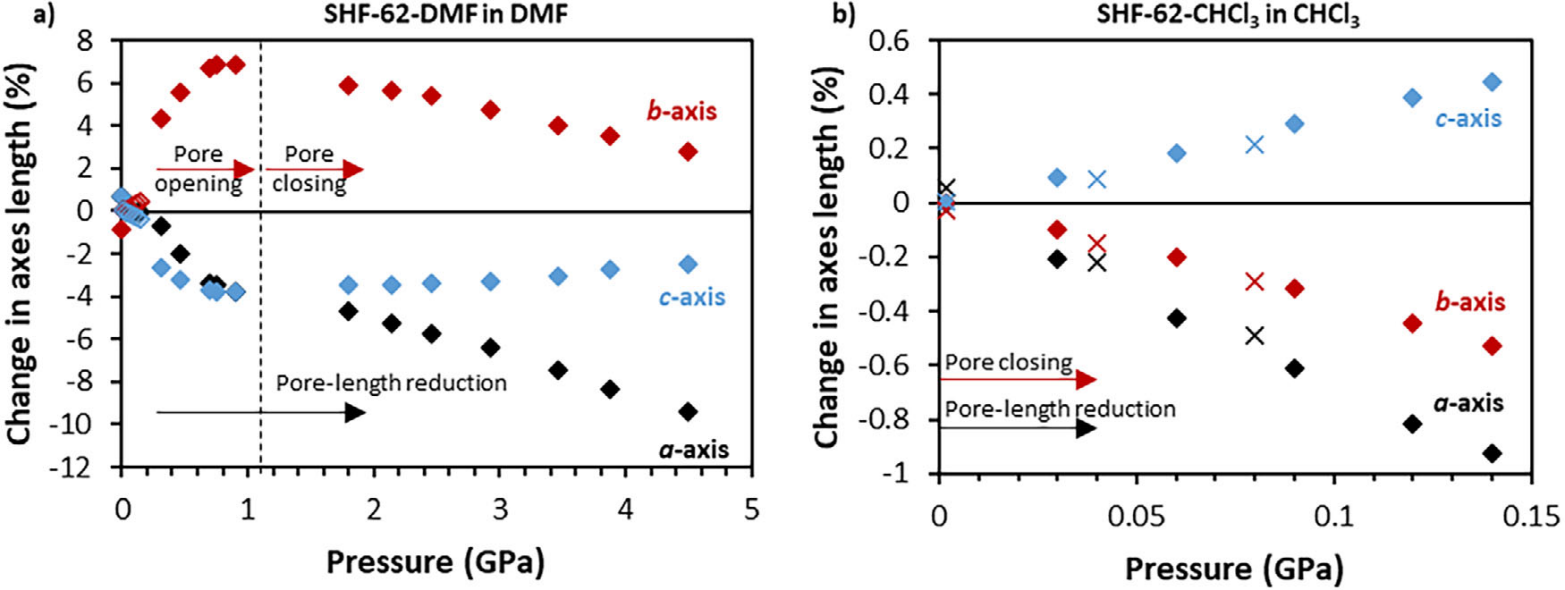
Changes in unit cell dimensions for a) **SHF‐62‐DMF** under pressure of penetrating medium DMF and b) **SHF‐62‐CHCl_3_** under pressure of CHCl_3_, respectively. In a), filled diamonds are data from DAC study, empty diamonds from SCC study. In b), diamond symbols represent data points during pressure increase and cross symbols represent data points during pressure reduction.

Complementary DAC pressure studies (*p* > 0.15 GPa) on a separate single crystal showed that the negative correlation of framework motions, involving pore‐length shortening with pore cross‐section expansion, continues at higher pressures, up to a maximum *b*‐axis length of 28.670(2) Å at 0.91 GPa (Δ*b* = +7.7%) (Figure 3a). This framework hyperexpansion represents the largest pore opening (*O *= 96%) observed for this family of frameworks with DMF content. Beyond 0.91 GPa, the effect of external pressure overcomes the pore expansion, and the pore begins to close, restoring the positive correlation between changes to pore length and pore cross‐section, as observed for the nonpenetrating medium FC‐70. Throughout this study, the crystallinity is retained, enabling SCXRD measurements beyond those accessible using FC‐70; the penetrating nature of the PTM increases the pore‐solvent content, which engenders increased stabilization against applied external pressure. The increase in pore‐solvent content is also inferred from the fact that even at the upper pressure of 4.5 GPa the pore cross‐section remains more open than the starting dimensions recorded at ambient pressure.

Overall, the deformations associated with the pore‐length and pore‐cross‐section flexible motions result in the unit cell volume initially *increasing* up to *p* = 0.32 GPa. In this pressure range, the pore opening more than compensates for the contraction in the pore length involving compression of the framework helices. Beyond this pressure, the unit cell volume decreases as the rate of expansion due to pore opening first decreases and then (at *p* > 0.91 GPa) reverses, such that both motions result in framework compression.

### Penetrating or Nonpenetrating Medium (CHCl_3_)?

CHCl_3_ is similar in molecular volume and bulk fluid packing density to DMF,^[^ ^60^ ^]^ and might be anticipated to act as a penetrating PTM as observed for DMF. X‐ray diffraction studies using CHCl_3_ as the PTM are traditionally very challenging due to the low freezing pressure of CHCl_3_ at ambient temperature (*p* ≈ 0.6 GPa)^[^ ^61^ ^]^ leading to a very high background in diffraction measurements. Although studies at higher pressures using the DAC are not feasible due to this restriction, full structural studies of **SHF‐62‐CHCl_3_** were possible over a range of pressures up to *p* = 0.14 GPa by using the SCC. (Figure 3b). In contrast to the study with DMF as the PTM, no opening of the framework resulted. Instead, both pore‐length shortening (Δ*a* ≈ −1.0%) and pore‐channel closing occurred (−Δ*b* > 0.5%; Δ*c* < +0.5%). This correlated behavior is consistent with that observed for both **SHF‐62‐CHCl_3_** and **SHF‐62‐DMF** when using the nonpenetrating PTM, FC‐70, suggesting that the ambient pressure structure for **SHF‐62‐CHCl_3_** cannot accommodate additional CHCl_3_ into the channels. Thus, CHCl_3_ is acting as a nonpenetrating PTM. The structural response of the framework was fully reversible on stepwise reduction of the pressure to 0.002 GPa (Figure 3b, Table S5), again consistent with the absence of (net) ingress of CHCl_3_ into the MOF pores during the pressure study.

### Structural Response to Pressure: Framework Dynamics

**Figure 4:**
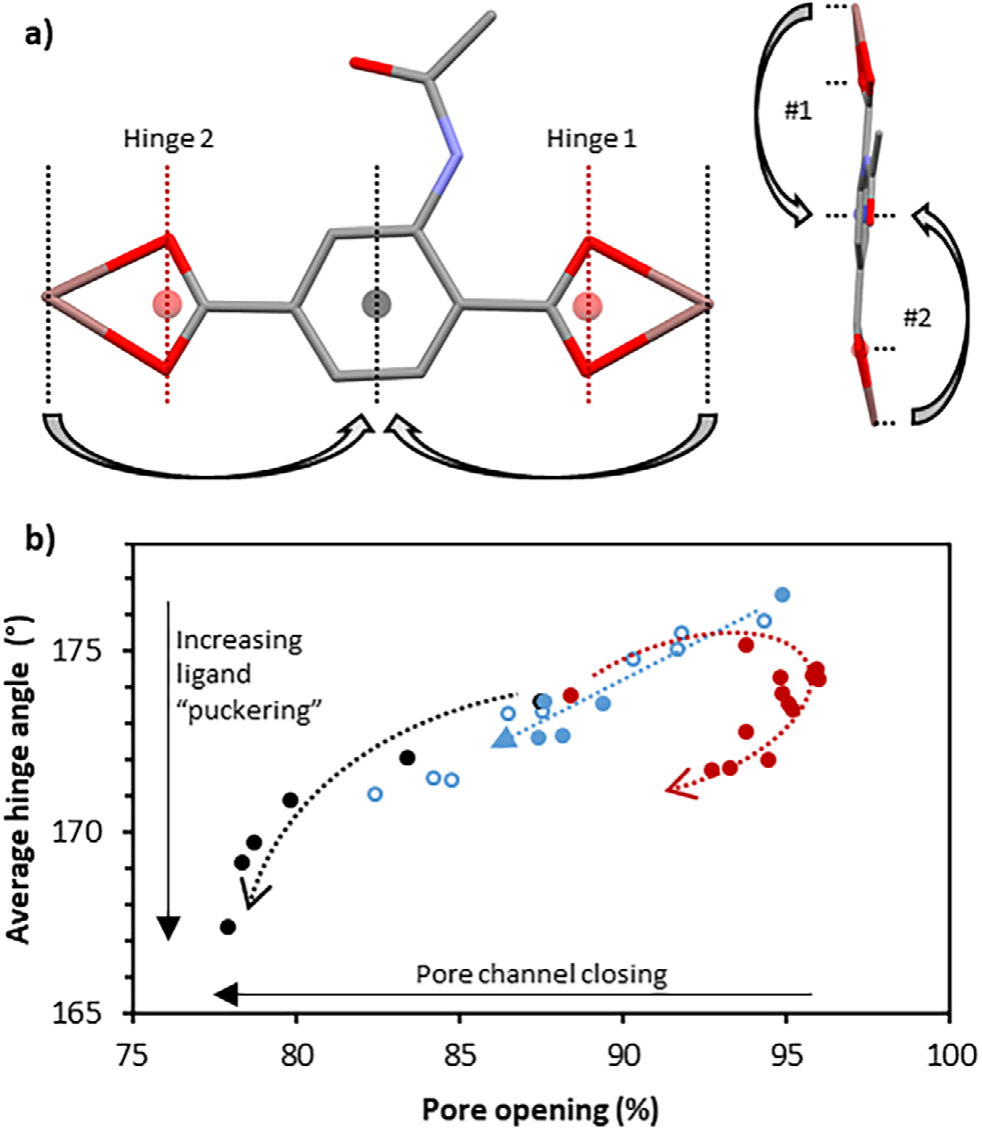
a) Framework ligand, BDC‐NHC(O)Me, shown linking two In centres with hinges about the carboxyl vectors represented. O⋯O centroids and the centroid of aromatic ring are defined and used for the In → O⋯O centroid → ring‐centroid angle. b) Changes to the average hinge angle in **SHF‐62‐DMF** and **SHF‐61‐CHCl_3_** as a function of the pore opening. This enables comparison between the dynamics upon activation/desolvation^[^ ^54^ ^]^ (unfilled, blue) and under compression for **SHF‐62‐CHCl_3_** in FC‐70 (filled, blue) and for **SHF‐62‐DMF** in FC‐70 (black) and DMF (red). Dotted lines are a guide to the pathway under increasing pressure.

In the previous study of the activation processes of this and closely related frameworks,^[^ ^53^, ^54^ ^]^ the two motions associated with framework dynamics (helix compression/elongation and pore closure/opening) have always been observed to act in a monotonically, although not necessarily linearly, correlated manner. Thus, upon activation to remove pore solvent, the framework helices compress, decreasing pore length, and pore channels close, decreasing pore cross‐section, and upon gas/solvent uptake, the helices elongate and the pore channels open. In the present study, we observe a significant deviation from this cooperative behavior for the first time. When using DMF as a penetrating medium, **SHF‐62‐DMF** undergoes compression of the framework helices throughout the pressure regime studied, whereas the pore channels initially open from *O* = 88 % to 96 % (0 < *p* < 1 GPa, Figure 4a) before closing to *O* = 93 % (*p* = 4.5 GPa). This demonstrates that flexible motions within this framework can operate independently (uncooperatively) as a function of stimulus (pressure, in this work), and therefore the motions can be said to be *decoupled*. Diffraction data were sufficiently complete to allow crystal structure refinement of the framework, enabling a molecular interpretation of the dynamic behavior.

We have previously established that the breathing motion of the MOF involves a hinge motion about the two carboxylate O⋯O vectors of the framework ligand (Figure 4a and Section S4.4.1).^[^ ^53^, ^54^ ^]^ We have mapped the hinge angles observed in these pressure studies alongside those observed during the CHCl_3_ desolvation pathway,^[^ ^54^ ^]^ enabling direct comparison between the two different environments (Figure 4b). There is a strong linear correlation between hinge angle and increasing desolvation, wherein the ligand angles become more acute (i.e., increased ligand puckering) with reducing pore content. Compression of **SHF‐62‐CHCl_3_** in FC‐70 follows this trend very well, showing that the application of pressure can mimic the desolvation pathway (Figure 4b, blue circles). For **SHF‐62‐DMF** compression in FC‐70, we are able to push the framework further, until a pore opening level of approximately 80%, below which the ligand deformation becomes more rapid (*p* > 0.5 GPa) leading to the deterioration of the crystal at *p* > 1.2 GPa (*O* = 78%) (Figure 4b, black filled circles). This case highlights that the solvent and its positions within the framework pores must play a role in the stabilization, under compression, of reduced pore openings until a point is reached where diffraction quality no longer enables reliable modelling. More generally, this shows that it may be possible to use pressure as a proxy stimulus for activation to understand the structural deformations at the onset of framework collapse for study of other MOFs that are unstable under activation.

Compression of **SHF‐62‐DMF** using DMF as a penetrating PTM shows a substantial departure from its structural behavior when using the nonpenetrating medium, FC‐70. We observed an initial increase in pore cross‐section (0 < *p* < 0.91 GPa) and the unit cell volume (0 < *p* < 0.32 GPa) on compression in DMF, which we attribute to penetration of the medium into the pores, in line with previous observations for other metal‐organic materials.^[^ ^25^, ^34^, ^52^ ^]^ Analyses of crystal structures show the impact of this increasing pore content on the framework itself. The framework pores at the start of the pressure series (*p* = 0.002 GPa) are not as fully open as observed when CHCl_3_ fills the pores (88% vs. 96%), which is consistent with there being different strengths of solvent‐framework interactions within the pore environments. Initially, the intake of DMF into **SHF‐62‐DMF** expands the pore cross‐section of the framework to dimensions similar to those of the framework characterized for **SHF‐62‐CHCl_3_** under ambient conditions. We have previously noted that **SHF‐62‐CHCl_3_** has more expanded pores than **SHF‐62‐DMF** under ambient conditions. Under such greater expansion the ligand O⋯O hinge angles approach linearity. After the structure has opened, the framework ligands then start to buckle upon further increase in pressure (decreasing O⋯O hinge angles at *p* > 1 GPa). A large pore opening (*O* = 93%) is maintained, however, indicating the retention of DMF in the pores.

One of the key aspects from our original study of **SHF‐62** activation was the substantial change in the orientation of the ligand as **SHF‐62** was desolvated. Approximately 50 % of ligand aromatic rings rotated (“flipped”) by ∼150° to avoid a steric clash of pendant amide methyl groups from neighboring ligands that are brought into close proximity through pore closure.^[^ ^54^ ^]^ Under compression, there is no evidence of such ring flipping, when using either nonpenetrating or penetrating PTMs. Thus, whereas ligand flipping behavior was observed when *O* < 87% during activation/desolvation, compression in FC‐70 distorts the framework well below this pore opening value but flipping is not observed. The most plausible explanation for the absence of this ligand flip upon framework compression under pressure is that the pore content is consistent throughout the pressure regime. This contrasts with the desolvation pathway during which solvent is lost, providing an increase in the available space for the ligand to change orientation and relocate the amide substituent within the pore. The retention of the solvent in the pores in the pressure studies therefore prevents reorientation of the ligands despite the methyl groups being brought into close proximity through compression in FC‐70 (C⋯C = 3.78 Å at *p* = ambient; C⋯C = 2.25 Å at *p* = 1.2 GPa for **SHF‐62‐DMF** in FC‐70). Mapping the direction of motion of the methyl groups (using the positions of modelled methyl carbon atoms) relative to one another as a function of increasing pressure shows that the groups exhibit little lateral motion to minimize steric clash when using DMF as the PTM. For both solvated frameworks (**SHF‐62‐DMF** and **SHF‐62‐CHCl_3_**) in FC‐70, however, the methyl‐methyl C⋯C distance decreases with increasing pressure, but the methyl group motions can be tracked along an arc that minimizes the steric clash between the two neighboring groups (Figure S14).

## Conclusion

**SHF‐62** is a highly flexible, diamondoid MOF that has been characterized along a continuum of structural response to pressure by single‐crystal X‐ray diffraction. Retention of crystallinity across a substantial pressure range makes it highly amenable to in situ diffraction study. This has enabled our tandem use of pressure cells (sapphire capillary cell (0.002–0.14 GPa) and diamond‐anvil cell (0.15–5 GPa)) in conjunction with high‐intensity synchrotron radiation to reveal nuances of structural evolution in this flexible framework material.

Direct compression using FC‐70, as a nonpenetrating pressure‐transmitting medium, resulted in analogous behavior in both solvated forms, **SHF‐62‐DMF** and **SHF‐62‐CHCl_3_**, with decreases in the *a*‐axis, characterizing a compression of the framework helices that run along the pore length parallel to this axis. In addition, the pore channels close (extent of pore opening, *O*, defined by changes to the *b*‐ and *c*‐axes), resulting in an overall decrease in unit cell volume of 10.7% (**SHF‐62‐DMF**) and 11.6% (**SHF‐62‐CHCl_3_**), up to maximum pressures of 1.2 and 1.4 GPa, respectively. For **SHF‐62‐DMF**, hypercompression of the pore channel from *O *= 86% to *O *= 78% was observed, extending the limits of flexibility in the system beyond those achievable through solvent loss at ambient pressure (*O *= 82%)^[^ ^54^ ^]^ upon framework activation.

Application of pressure to **SHF‐62‐DMF** in DMF, a framework‐penetrating PTM, resulted in markedly different behavior. In the SCC, an increase of the unit cell volume, with concurrent opening of the pore channel, indicated DMF ingress upon increasing pressure. This phenomenon was amplified when using the DAC pressure regime, resulting in hyperexpansion of the framework to *O*_max _= 96 %, after which (*p* > 1 GPa) external pressure overcomes framework resistance to pore closure and *O* decreases. Remarkably, monotonic pore‐length contraction (Δ*a* < 0) occurs both during pore‐opening (Δ*O* > 0) and pore‐closing (Δ*O* < 0) ranges, demonstrating unprecedented decoupling of these framework motions for the first time. Thus, one motion compresses the framework while, simultaneously, the other can either expand or compress. Such decoupling of motions is not observed when using CHCl_3_ as the PTM, a study only feasible through access to pressures below the freezing pressure of CHCl_3_, by using the SCC. No (net) CHCl_3_ penetration of the pores is observed, highlighting once more that dynamic behavior can have solvent dependence in this family of MOFs.^[^ ^53^, ^54^ ^]^

**Figure 5:**
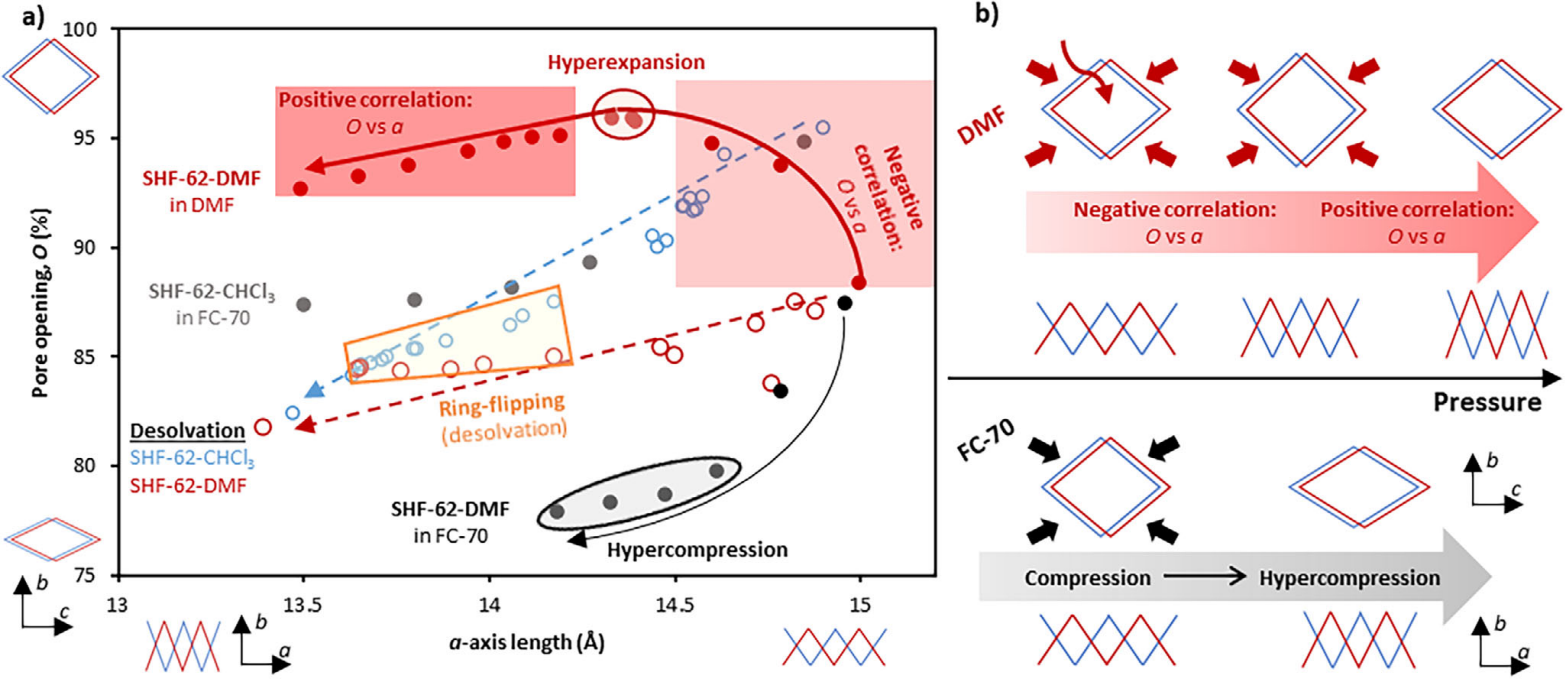
a) Overview of changes in unit cell dimensions, with contextualized schematics of framework motions. Activation studies (previous work)^[^ ^54^ ^]^ are represented by empty red (**SHF‐62‐DMF**) and blue (**SHF‐62‐CHCl_3_**) circles, and pressure studies (this work) by filled circles. Arrows indicate direction of activation (dotted lines) or presurization (solid lines). b) Schematic of different flexible motions from different PTMs. Note: sketched representations of flexible motions indicate direction of motion only and are not to scale.

This study represents a significant step toward understanding the structural response to pressure of a highly flexible MOF, **SHF‐62**, using both framework penetrating and nonpenetrating media, as summarized in Figure 5. The approach of combining SCC and DAC studies provides a comprehensive range of accessible pressures with which to characterize this framework response. Considering the global body of data now available for this MOF family, we have demonstrated the nuanced structural behavior that the use of pressure, as a new structure‐perturbing stimulus, can elucidate. This tandem pressure‐cell approach is well suited to the study of structural flexibility in a range of soft, pressure‐responsive materials. We anticipate that this (and other) combination(s) of pressure cells will expand the working knowledge of responsive materials and enable the rational design of new functional materials.

## Supporting Information

Supporting Information is available and includes full synthetic details, materials characterization, unit cell parameters, and structural analyses. Crystal structure data have been deposited with the CSD and can be freely accessed online via by the joint Cambridge Crystallographic Data Centre and Fachinformationszentrum Karlsruhe Access Structures service at https://www.ccdc.cam.ac.uk/structures/. CSD deposition numbers 2402822‐2402882 are listed alongside the corresponding crystal structure in the Supporting Information. The authors have cited additional references within the Supporting Information.^[^ ^62^, ^63^, ^64^, ^65^, ^66^, ^67^, ^68^, ^69^, ^70^, ^71^, ^72^, ^73^ ^]^

## Author Contributions

E.J.C. and L.B. conceptualized the project. E.J.C. and T.M.R. synthesized all materials. D.J.A., E.J.C., T.M.R., C.J.M., M.R.W., M.R.W., S.A.M., I.D.H.O. and L.B. recorded experimental data at Diamond Light Source. D.J.A., E.J.C., and T.M.R. performed postprocessing and data analysis with support from S.A.M., M.R.W., M.R.W., I.D.H.O. and L.B. A.J.F., T.D., I.D.H.O., S.A.M., and L.B. provided supervisory support and funding acquisition. D.J.A. drafted the manuscript with support from T.M.R., I.D.H.O., and L.B. All authors contributed to writing — review and editing. L.B. coordinated the project.

## Conflict of Interests

The authors declare no conflict of interest.

## Supporting information



Supporting Information

Supporting Information

## Data Availability

Data underpinning this publication are openly available from the University of Strathclyde KnowledgeBase at https://doi.org/10.15129/acde92cc‐96ae‐4234‐ace4‐2164a407e5d1.
