# Supplementary material for: Decoupled MOF Breathing: Pressure‐Induced Reversal of Correlation Between Orthogonal Motions in a Diamondoid Framework
Source: Angew Chem Int Ed Engl. 2025 May 15;64(27):e202504297. doi: 10.1002/anie.202504297 (PMC12207364; doi:10.1002/anie.202504297)
Supplement: Supplementary file 2 — Supporting Information [file ANIE-64-e202504297-s001.pdf]

Supplementary Information

Decoupled MOF Breathing: Pressure-Induced Reversal of Correlation between Orthogonal Motions in a Diamondoid Framework

David J. Ashworth,[1,2] Elliot J. Carrington,[3] Thomas M. Roseveare,[3] Charles J. McMonagle,[4] Martin R. Ward,[2] Ashleigh J. Fletcher,[1] Tina Düren,[5] Mark R. Warren,[6] Stephen A. Moggach,[7] Iain D. H. Oswald[2]* and Lee Brammer[3]*

*corresponding authors email: iain.oswald@strath.ac.uk; lee.brammer@sheffield.ac.uk

[1]Department of Chemical and Process Engineering, University of Strathclyde, Montrose Street, Glasgow, G1 1XJ, UK.

[2]Strathclyde Institute of Pharmacy & Biomedical Sciences (SIPBS), University of Strathclyde, 161 Cathedral Street, , Glasgow, G4 0RE, UK.

[3]Department of Chemistry, University of Sheffield, Brook Hill, Sheffield, S3 7HF, UK.

[4]The European Synchrotron Research Facility, 71 Avenue des Martyrs, 38000 Grenoble, France.

[5]Centre for Integrated Materials, Processes and Structures and Department of Chemical Engineering, University of Bath, Claverton Down, Bath, BA2 7AY, UK.

[6]Diamond Light Source, Harwell Science and Innovation Campus, Didcot, OX11 0DE, UK.

[7]Centre for Microscopy, Characterisation and Analysis, and School of Molecular Science, The University of Western Australia, 35 Stirling Highway, Crawley, Perth 6009, Australia.

Table of Contents

S1. Materials and methods S3

S1.1 Materials S3

S1.2 Methods S3

S1.3 High-pressure single-crystal X-ray diffraction studies using synchrotron radiation S3

S2. MOF syntheses and characterisation S5

S2.1 Syntheses S5

S2.2 Characterisation S5

S3 Calculating pore channel opening S8

S4 High-pressure X-ray diffraction data S9

S4.1 Unit cell parameters (and CSD deposition numbers) S9

S4.2 Sapphire capillary cell (SCC) studies - reversible response to pressure S11

S4.3 Fitting equations of state S13

S4.4 Structural analyses S14

S5 References S17

S1. Materials and methods

S1.1 Materials

All reagents and solvents were used as supplied by the commercial supplier without further purification, other than $CHCl_3$: $InCl_3$ (Fluorochem, 99.9 %), amino-1,4-benzenedicarboxylic acid (Fisher Scientific, 99 %), acetic anhydride (Sigma Aldrich, 99 %), DMF (VWR, 99 %), FC-70 (Fluorinert, 99 %). $CHCl_3$ dried according to the method of Grubbs[S1] was used for **SHF-62-CHCl$_3$** synthesis and storage.

S1.2 Methods

Powder X-ray diffraction. MOF samples were removed from solvent and allowed to dry in air, before being lightly ground to a microcrystalline powder and packed into a 0.7 mm borosilicate capillary. X-Ray powder diffraction patterns were collected using a Bruker D8 Advance powder diffractometer equipped with a Cu K_α source (λ = 1.5418 Å) operating at 40 kV and 40 mA in Debye-Scherrer geometry. The instrument was fitted with an energy-dispersive LYNXEYE detector.

^{1}H NMR spectroscopy. ~10 mg MOF were filtered from solvent and added to 0.75 mL d_6-DMSO. 10 µL of DCl (35 %) in D_2O was then added to "digest" the MOF. The solution was added to an NMR tube and used without neutralisation. NMR spectra were recorded on a Bruker Advance DPX 400 MHz spectrometer. ^{1}H chemical shifts are reported in ppm on the δ scale and were referenced to the residual solvent peak. All coupling constants are reported in Hz.

S1.3 High-pressure single-crystal X-ray diffraction studies using synchrotron radiation

Diamond-Anvil Cell (DAC). Single crystals were loaded into Merrill-Bassett DACs with a half-opening angle of 40 °, composed of Boehlar Almax diamonds with 600 µm culet diamond anvils, a tungsten gasket and tungsten carbide backing plates. A small ruby chip was also loaded into each cell as an internal pressure calibrant to calculate the internal pressure by using the pressure-dependent fluorescence of the ruby. Either DMF, $CHCl_3$ or Fluorinert FC-70 ($N(C_5F_{11})_3$) was used as the pressure transmitting medium (PTM).

Sapphire Capillary Cell (SCC). Single crystals were either attached to a carbon fibre using Araldite® glue (FC-70 as PTM) or mechanically held in place by wedging the crystal between split ends of a carbon fibre (DMF or $CHCl_3$ as PTM) and loaded inside a SCC, which has been previously described.[S2] Pressure was increased to 20 bar in order for residual air in the capillary to dissolve into the PTM, thereby filling the cell, which provided the loading pressure for the initial X-ray data collection.

High-pressure single-crystal synchrotron X-ray diffraction data collection. Single-crystal X-ray diffraction data using both SCCs and DACs were collected at beamline I19 (EH2),[S3] Diamond Light Source. Data were collected at a wavelength of 0.4859 Å using a Pilatus 300 K (most data) or Eiger2 4M CdTe (SCC, **SHF-62-CHCl$_3$** in $CHCl_3$ and **SHF-62-DMF** in FC-70) detector. Data were collected at the ambient temperature (approximately 25 °C) without further control of the sample temperature.

X-ray diffraction data processing and crystal structure refinement. Most X-ray data were processed with *CrysAlis^{Pro} (*version16)[S4] including empirical absorption correction using spherical harmonics, implemented in SCALE3 ABSPACK scaling algorithm, using a data rejection criterion based on a DAC half-opening angle of 38 °. For the **SHF-62-CHCl$_3$** in $CHCl_3$ (SCC) dataset, data were processed using *Xia2* software,[S5] with an empirical absorption correction applied using a model for crystal shape derived from a summation of spherical harmonic functions, performed with *DIALS* 3.8.2-1-G50706578e-release.

All crystal structures were solved and refined against F^2 values using the program *SHELXL*,[S6] accessed within the *Olex2* program.[S7] Crystallographic models of atomic positions taken from previously published structures (**SHF-62-DMF** CSD Refcode AXINED and **SHF-62-CHCl$_3$** CSD Refcode AXIMEC)[S8] were used as the starting point for refinements of structures at ambient pressure, after which the starting model for structures at sequential increasing pressure points used the atomic positions of the model from previous pressure. All non-hydrogen atoms were refined anisotropically except those of the pendent amide functional group (-NC(=O)OMe), which were modelled isotropically.

Crystallographic restraints and constraints were applied to some structure models, where necessary. Positions of hydrogen atoms were calculated with idealised geometries and refined using a riding model with isotropic displacement parameters fixed in magnitude relative to the attached atoms.

Some previous structural models contained modelling of the phenyl rings and attached pendant amides as disordered over two positions (phenyl rotation < 20°). High pressure (DAC) data did not support this due to the reduced data quality resulting from limited opening angle of the DACs, hence a single position was modelled, resulting in atom positions with slightly higher thermal motion. The $Me_2NH_2^+$ cation was modelled in some cases (data from SCC) with the nitrogen atom disordered over two positions, related in position by the local crystallographic 2-fold symmetry. We concluded that this was the best model in those cases as the alternative model with the nitrogen atom located at a single position coincident with the crystallographic 2-fold axis resulted in residual electron density on either side of the nitrogen atom. For the studies conducted in the DAC (SHF-62-DMF in DMF), the cation was modelled as a rigid body, placed close to the maximum residual density in the difference map, followed by refinement of the position and orientation of the rigid body. This was necessary due to the paucity of data, as is typical for high-pressure SCXRD measurements in DACs, and also reflects the fact that the modelling of the cation and solvent was challenging even in our earlier study conducted at low temperature study and ambient pressure.[S11] The crystal structure at the highest pressure point in this series (p = 4.49 GPa) indicates a less optimum positioning of the cation but the resulting structure is where the rigid body refined from many trials of cation placement. For the DAC data in which FC-70 was used as the PTM, we modelled two positions of the cation based on the size of the U_{iso} parameter which we felt better models the data.

Full details of data collection, crystal data and crystal structure refinements can be found in the deposited files in CIF format.

Inherent limitations of high-pressure single-crystal X-ray diffraction (HP-SCXRD) studies

Although single-crystal X-ray diffraction remains the “gold standard” in crystal structure determination, in high-pressure crystallography limitations in data quality, data completeness and detail accessible from structural models are inherent from use of the sample cells required to apply hydrostatic pressure to the crystal. Thus, for example, restrictions in aperture for Diamond-anvil cells (DACs) noted above, have limited the data completeness in our studies to approx. 60%,[S10] which is typical for a HP-SCXRD study, but differs from the 95-100% completeness that is common for conventional SCXRD under ambient conditions. Data processing (indexing and integration) must also account for the effect of diffraction from the diamond crystals and from the tungsten gaskets used in assembly and function of the cells and the enhanced background scattering from the liquid pressure-transmitting medium (PTM) that surrounds the crystal.[S11] The sapphire-capillary cells (SCCs) have fewer restrictions, but still require transmission of incident and diffracted X-rays through the single-crystal sapphire capillary and the liquid PTM.[S2] This adds appreciably to the background signal and the sample diffraction pattern is contaminated by diffraction from the sapphire crystal.

S2. MOF syntheses and characterisation

S2.1 Syntheses

SHF-61 was synthesised, initially in its DMF-solvated form (**SHF-61-DMF**) in accordance with our previous methodology.[S8] In a typical synthesis, $(Me_2NH_2)[In(BDC\text{-}NH_2)_2]\cdot 2(DMF)$ (BDC=1,4-benzenedicarboxylate) (**SHF-61-DMF)** was prepared by solvothermal methods from reaction between $InCl_3$ (0.1106 g, 0.5 mmol) and amino-1,4-benzenedicarboxylic acid (0.092 g, 0.5 mmol) in DMF (10 mL). Reactants and solvent were sealed inside a Teflon-lined steel autoclave and heated to 110 °C for 24 hrs, then cooled at 0.1 °C min^{-1} to 25 °C, and washed with fresh DMF, yielding **SHF-61-DMF**, as confirmed by bulk characterisation (PXRD) consistent with that previously reported.[S8]

SHF-61-DMF (100 mg, 0.2 mmol) was then reacted with five equivalents of acetic anhydride (100 µL, 1 mmol) in dry chloroform (0.6 mL) in a sealed vessel at 55 °C in an oven for 24 hrs, cooled at 0.1 °C min^{-1} to 25 °C, then washed with dry $CHCl_3$ to yield $(Me_2NH_2)[In(BDC\text{-}NHC(O)Me)_2]\cdot CHCl_3$ (**SHF-62-CHCl₃**). This was solvent exchanged with DMF for 5 days (DMF changed daily) to yield $(Me_2NH_2)[In(BDC\text{-}NHC(O)Me)_2]\cdot CHCl_3$ (**SHF-62-DMF**).[S9] Bulk characterisation by PXRD and NMR spectroscopy is described in Section S2.2.

S2.2 Characterisation

PXRD and Pawley fitting. Phase purity of the MOFs was confirmed using PXRD. PXRD patterns were collected at room temperature on the in-house diffractometer (Section S1.2.) with an angular range of 3° ≤ 2θ ≤ 60°, step size 0.02 and collection time of 1 s $step^{-1}$.

Diffraction patterns were analysed by Pawley refinement,[S12] using *TOPAS-Academic* version 7.25[S13-S15] in conjunction with jEdit (Figures S1 and S2; Table S1). The equations below define R_{wp} and R_{wp}', the indices of agreement between calculated and experimental diffraction patterns:

$$R_{wp} = \sqrt{\frac{\Sigma[w(Y_{obs}-Y_{calc})^2]}{\Sigma[wY_{obs}{}^2]}} \qquad R_{wp}' = \sqrt{\frac{\Sigma[w(Y_{obs}-Y_{calc})^2]}{\Sigma[w(Y_{obs}-bkgr)^2]}}$$

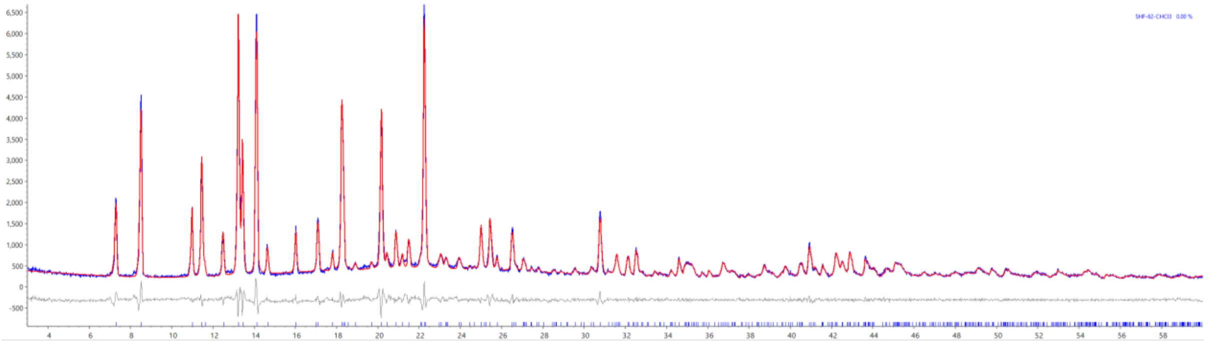

Figure S1. PXRD pattern of **SHF-62-CHCl₃**, with Pawley fit, illustrating the observed (blue) and calculated (red) diffraction patterns, with the difference plot $[I_{obs}\text{-}I_{calc}]$ (grey) (3.0 ≤ 2θ ≤ 60.0 °; d_{min} = 0.89 Å). Blue tick lines denote calculated peak positions.

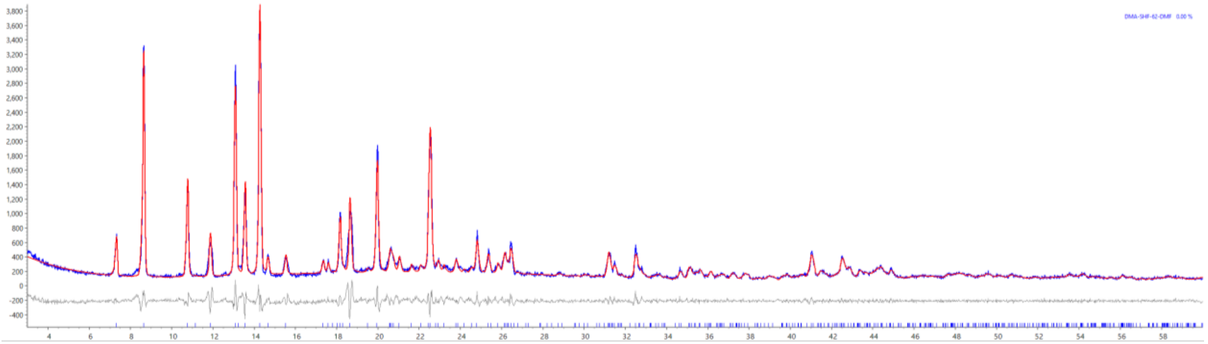

Figure S2. PXRD pattern of **SHF-62-DMF**, with Pawley fit, illustrating the observed (blue) and calculated (red) diffraction patterns, with the difference plot [I_{obs}-I_{calc}] (grey) (3.0 ≤ 2θ ≤ 60.0 °; d_{min} = 0.89 Å). Blue tick lines denote calculated peak positions.

Table S1. Summary of PXRD Pawley refinement fitting data.

MOF	*a* (Å)	*b* (Å)	*c* (Å)	*V* (Å³)	R_{wp}	R_{wp}'
**SHF-62-CHCl₃**	14.8618(6)	28.284(1)	30.437(1)	12794.3(9)	0.0752	0.1427
**SHF-62-DMF**	14.961(1)	26.770(2)	31.693(3)	12693(2)	0.1106	0.2065

^{1}H NMR Spectroscopy. A small amount of MOF was "digested" by adding 5-10 µL of 35% DCl in D_2O to ~3 mg MOF in d_6-DMSO, to: (1) quantify the extent of post-synthetic modification of the pendant amine groups to form methylamide groups in **SHF-62-CHCl₃** (Figure S3), (2) establish complete solvent exchange from $CHCl_3$ to DMF in **SHF-62-DMF** (Figures S3 and S4), and (3) to confirm sample purity through the absence of other organic components. Acidic protons are not observed due to facile exchange with deuterium, which also can exchange with the amide proton (H_5) resulting in underrepresentation of this proton environment.

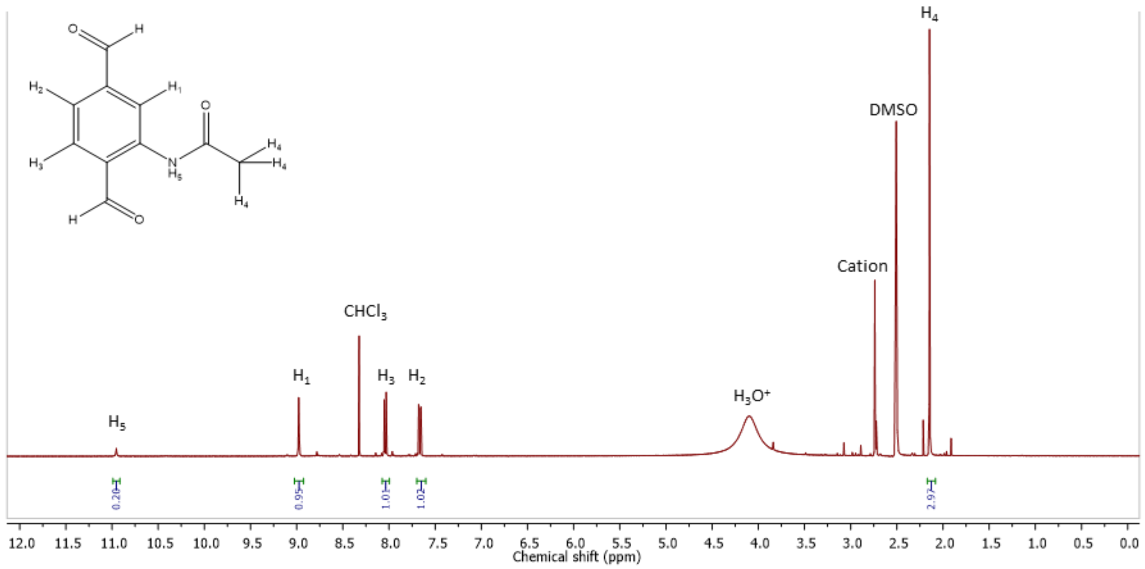

Figure S3. ^{1}H NMR spectrum of **SHF-62-CHCl₃** after digestion using DCl/D_2O in d_6-DMSO; Cation = $Me_2NH_2^+$.

The spectrum in Figure S3 shows no evidence of the unfunctionalised aminobenzenedicarboxylic acid, indicating 100 % post-synthetic modification of the ligand has been achieved. Equivalent phenyl proton environments for aminobenzenedicarboxylic acid would be observed at δ 7.74 ppm (s), 7.47 ppm (d) and 7.91 ppm (d) for H_1-H_3, respectively.[S8]

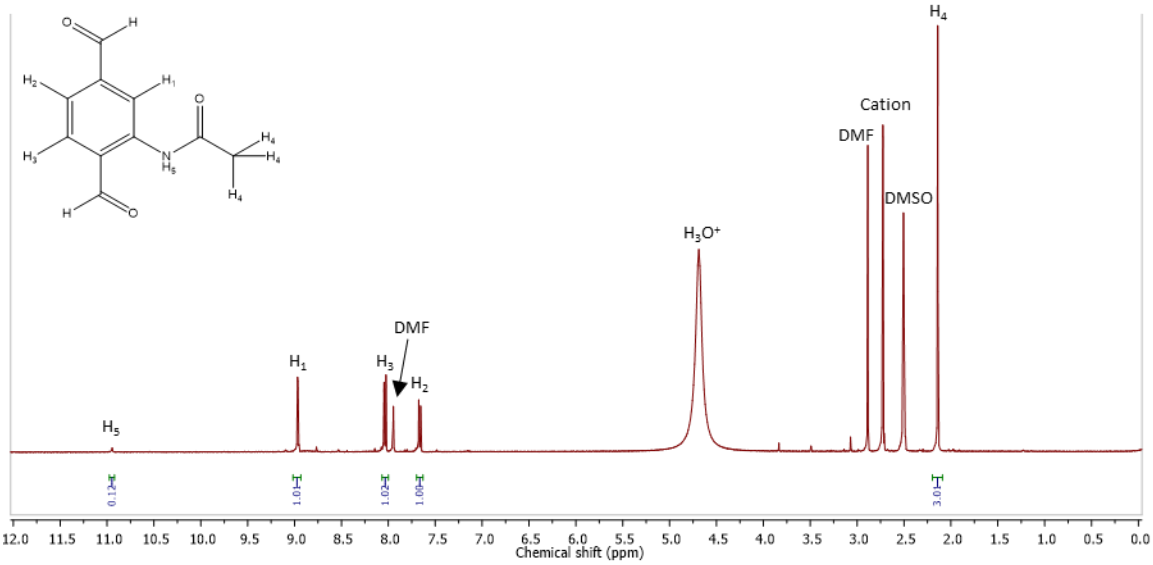

Figure S4. ^1H NMR spectrum of **SHF-62-DMF** after digestion using DCl/D_2O in d_6-DMSO; Cation = $Me_2NH_2^+$.

The spectrum in Figure S4 shows no evidence of C<u>H</u>Cl$_3$ (δ 8.33 ppm, see Figure S3), confirming complete solvent exchange to DMF.

Neither spectrum (Figure S3 or S4) shows significant unassigned peaks, thereby confirming sample purity.

S3 Calculating pore channel opening

There is not a consistent measure of pore opening that can be defined simply from interatomic angles because the helices within the framework that run parallel to the length of the pore channel can expand and contract (change in the *a*-axis length), changing interatomic angles without necessarily being related in a consistent manner to changes in the wine-rack like motif associated with pore opening.

Instead, the pore channel can be described in terms of pore opening (*O*). The crystallographic *b*- and *c*-axes run parallel to the channel cross-section, and so can be used to describe the opening.

The pore-opening, *O*, can be quantified (Figure S5) as a projection of the pore channel onto a 2D plane parallel to the *bc*-plane by using the trigonometric function:

$$O\ (\theta) = tan^{-1}(b/c)$$

$$O\ (\%) = \frac{tan^{-1}(b/c)}{45\ ^\circ} \times 100$$

O_{max} = 100 %, when $b = c$.

O_{min} = 0 %, where the pore is completely collapsed to zero cross-sectional area ($b = 0$, $c = c_{max}$). Note, this is not a physically achievable geometry.

For example:

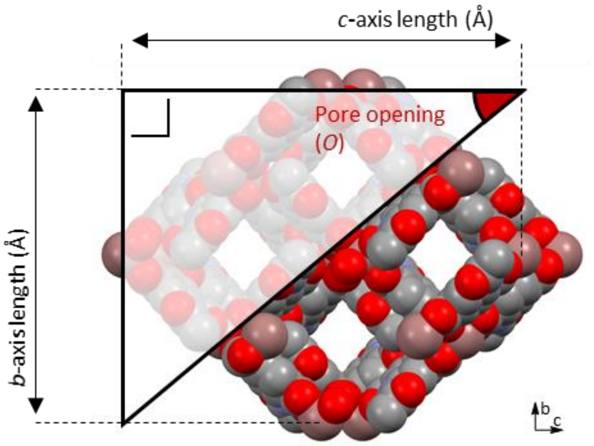

b = 26.598(4) Å
c = 31.962(3) Å

$$O\ (^\circ) = \tan^{-1}\left({}^{b}/_{c}\right) = 39.77\ ^\circ$$

$$O\ (\%) = \frac{\tan^{-1}\left({}^{b}/_{c}\right)}{45\ ^\circ} \times 100 = \frac{39.77\ ^\circ}{45\ ^\circ} \times 100\ = 88\ \%$$

Figure S5. Example calculation of pore opening (%), in this case for *p* = initial for **SHF-62-DMF** in DMF (DAC).

To provide context for the current study, use of this method to calculate *O* for the previously published activation pathways for **SHF-62-CHCl$_3$** shows $O_{solvated}$ = 96 %, $O_{desolvated}$ = 82 %, and for **SHF-62-DMF** shows $O_{solvated}$ = 87 %, $O_{desolvated}$ = 82 %.[S8]

S4 High-pressure X-ray diffraction data

All CIFs have been deposited with the CCDC. These data can be obtained free of charge *via* https://www.ccdc.cam.ac.uk/structures/ and therefore tables of full structural details are not provided here. The main manuscript presents unit cell parameters as changes, relative to the respective unit cells at ambient pressure. For completeness, a summary of the absolute values for unit cell parameters *a*, *b*, *c* and volume are provided in Tables S2-S5 for ease of any further processing of data, such as calculation of % change, normalised changes, *etc*.

S4.1 Unit cell parameters

Table S2. Summary of unit cell parameters for **SHF-62-DMF** in FC-70.

Pressure (GPa)	*a* (Å)	*b* (Å)	*c* (Å)	Volume (Å^3)	CSD Deposition no.
**SCC study**					
0.002	14.8438(4)	26.1478(7)	32.1175(6)	12465.8(5)	2402876
0.02	14.8296(4)	26.0026(7)	32.2294(6)	12427.9(5)	2402872
0.04	14.8138(4)	25.8970(7)	32.3070(6)	12394.0(5)	2402873
0.06	14.7964(4)	25.7840(7)	32.3861(6)	12355.7(5)	2402878
0.08	14.7781(4)	25.6930(7)	32.4522(6)	12321.9(5)	2402881
0.1	14.7617(3)	25.6131(6)	32.5084(6)	12291.2(5)	2402882
**0.12**	14.7453(3)	25.5326(7)	32.5639(6)	12259.8(5)	2402874
0.08	14.7695(3)	25.7361(7)	32.4149(6)	12321.2(5)	2402871
0.04	14.7992(14)	25.9233(13)	32.2499(11)	12372.5(14)	unit cell only
0.002	14.8230(4)	26.1769(7)	32.0896(6)	12451.4(6)	2402875
**DAC study**					
0	14.9588(18)	26.386(2)	32.1633(19)	12695(2)	2402844
0.21	14.785(2)	25.322(3)	32.953(2)	12337(3)	2402845
0.48	14.613(3)	24.341(4)	33.619(3)	11958(3)	2402843
0.67	14.474(5)	24.001(7)	33.759(4)	11728(5)	2402841
0.88	14.324(8)	23.864(10)	33.769(7)	11543(8)	2402842
1.18	14.181(11)	23.685(15)	33.748(11)	11335(12)	2402840

Table S3. Summary of unit cell parameters for **SHF-62-CHCl$_3$** in FC-70.

Pressure (GPa)	*a* (Å)	*b* (Å)	*c* (Å)	Volume (Å^3)	CSD Deposition no.
**SCC study**					
0.00205	14.8905(3)	28.3334(5)	30.4700(4)	12855.3(4)	2402857
0.02	14.8564(3)	28.2886(5)	30.5119(5)	12823.1(4)	2402854
0.04	14.8239(3)	28.2372(5)	30.5552(5)	12790.0(4)	2402862
0.06	14.7861(4)	28.1799(6)	30.6053(5)	12752.4(5)	2402867
0.0799	14.7482(4)	28.1171(7)	30.6551(6)	12711.9(5)	2402870
0.1	14.7052(5)	28.0451(8)	30.7080(6)	12664.3(6)	2402858
**0.12**	14.6631(6)	27.9739(9)	30.7670(7)	12619.4(7)	2402859
0.08	14.7197(5)	28.0534(8)	30.6931(7)	12674.4(7)	2402861
0.04	14.7789(5)	28.1327(8)	30.6219(7)	12731.7(6)	2402855
0.0019	14.8368(5)	28.2000(7)	30.5597(6)	12786.1(6)	2402860
**DAC study**					
0	14.849(2)	28.2807(17)	30.6748(17)	12882(2)	2402823
0.34	14.269(10)	26.903(5)	31.826(5)	12217(9)	2402824
0.59	14.058(13)	26.621(7)	32.102(6)	12014(12)	2402825
0.96	13.798(16)	26.397(11)	32.134(9)	11704(15)	2402826
1.40	13.50(3)	26.29(2)	32.100(17)	11391(27)	2402822

Table S4. Summary of unit cell parameters for **SHF-62-DMF** in DMF.

**Pressure (GPa)**	***a* (Å)**	***b* (Å)**	***c* (Å)**	**Volume (Å^3)**	**CSD Deposition no.**
**SCC study**					
0.002	14.8937(7)	26.8484(16)	31.7555(15)	12698.1(11)	2402866
0.03	14.8899(6)	26.8932(14)	31.7039(12)	12695.5(9)	2402864
0.06	14.8757(16)	26.9756(15)	31.6398(13)	12696.5(10)	2402869
0.09	14.8628(6)	27.0883(14)	31.5405(12)	12698.4(10)	2402865
0.12	14.8538(6)	27.2149(12)	31.4329(11)	12706.6(9)	2402863
**0.14**	14.8482(6)	27.2827(12)	31.3771(10)	12710.9(8)	2402880
0.0803	14.8874(6)	27.2429(12)	31.4053(10)	12737.3(9)	2402879
0.0399	14.9061(6)	27.1766(14)	31.4564(12)	12742.9(10)	2402877
0.0021	14.9250(6)	27.1460(15)	31.4848(12)	12756.2(10)	2402868
**DAC study**					
0	14.996(8)	26.598(4)	31.962(3)	12748(7)	2402833
0.32	14.785(4)	28.002(3)	30.898(2)	12792(4)	2402831
0.47	14.600(4)	28.315(3)	30.725(2)	12702(3)	2402836
0.70	14.392(4)	28.610(2)	30.573(2)	12589(3)	2402832
0.76	14.382(4)	28.656(3)	30.555(2)	12593(4)	2402838
0.91	14.327(4)	28.670(2)	30.549(2)	12548(4)	2402828
1.80	14.191(6)	28.400(3)	30.649(3)	12352(5)	2402830
2.14	14.114(6)	28.344(3)	30.637(3)	12256(5)	2402835
2.45	14.038(6)	28.279(3)	30.667(3)	12174(5)	2402839
2.92	13.942(7)	28.098(3)	30.686(3)	12022(6)	2402837
3.46	13.781(8)	27.904(4)	30.787(4)	11839(7)	2402834
3.88	13.644(10)	27.780(4)	30.879(5)	11704(9)	2402829
4.49	13.492(11)	27.586(6)	30.950(7)	11520(10)	2402827

Table S5. Summary of unit cell parameters for **SHF-62-CHCl$_3$** in CHCl$_3$.

**Pressure (GPa)**	***a* (Å)**	***b* (Å)**	***c* (Å)**	**Volume (Å^3)**	**CSD Deposition no.**
**SCC study**					
0.002	14.82490(13)	28.3579(2)	30.3971(2)	12779.03(17)	2402847
0.03	14.79350(14)	28.3295(2)	30.4246(2)	12750.72(18)	2402853
0.06	14.76120(16)	28.3006(2)	30.4526(3)	12721.7(2)	2402846
0.09	14.73410(16)	28.2681(3)	30.4851(3)	12697.2(2)	2402849
0.12	14.70340(19)	28.2299(3)	30.5141(3)	12665.7(2)	2402856
**0.14**	14.6864(2)	28.2063(3)	30.5299(3)	12647.0(3)	2402850
0.08	14.7516(2)	28.2750(3)	30.4621(3)	12705.8(3)	2402848
0.04	14.7917(2)	28.3142(3)	30.4218(3)	12741.1(3)	2402852
0.002	14.8318(2)	28.3462(3)	30.3952(4)	12778.9(3)	2402851

S4.2 Sapphire capillary cell (SCC) studies - reversible response to pressure

In the manuscript, SCC data are presented solely as measurements of increasing pressure, for clarity, in Figures 2-5. Here, in Figures S6-S9, the changes in unit cell axes lengths are presented for both increasing and then decreasing pressures.

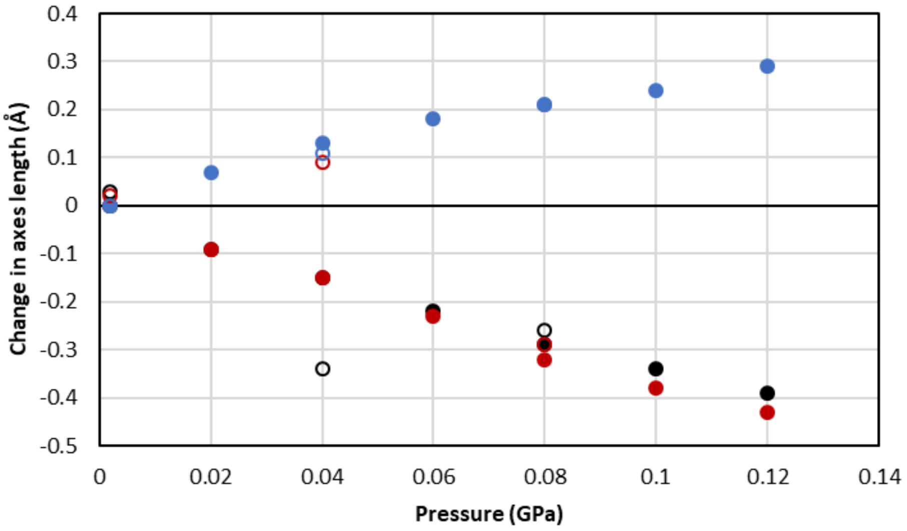

Figure S6. Changes in unit cell dimensions for **SHF-62-DMF** under pressure of FC-70 in the SCC. Filled symbols correspond to measurements made upon increasing pressure; empty symbols correspond to decreasing pressure. *a*-axis is black, *b*-axis is red, *c*-axis is blue. We note that data quality at 0.04 GPa in the decreasing pressure sequence was significantly reduced compared to other measurements.

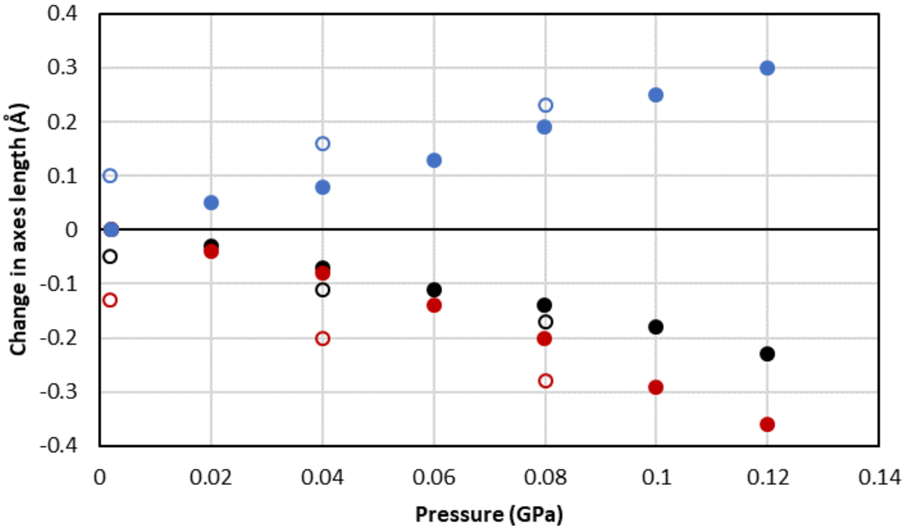

Figure S7. Changes in unit cell dimensions for **SHF-62-$CHCl_3$** under pressure of FC-70 in the SCC. Filled symbols correspond to measurements made upon increasing pressure; empty symbols correspond to decreasing pressure. *a*-axis is black, *b*-axis is red, *c*-axis is blue.

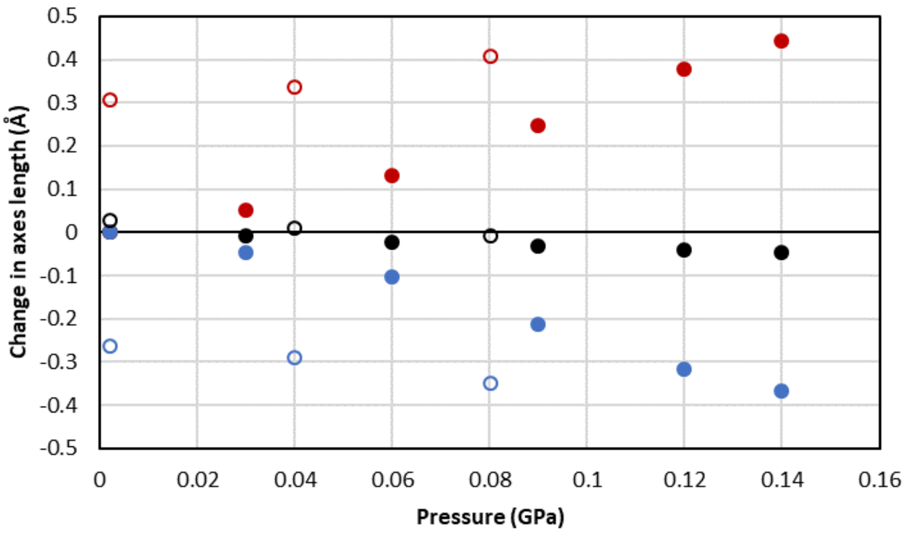

Figure S8. Changes in unit cell dimensions for **SHF-62-DMF** under pressure of DMF in the SCC. Filled symbols correspond to measurements made upon increasing pressure; empty symbols correspond to decreasing pressure. *a*-axis is black, *b*-axis red, *c*-axis is blue.

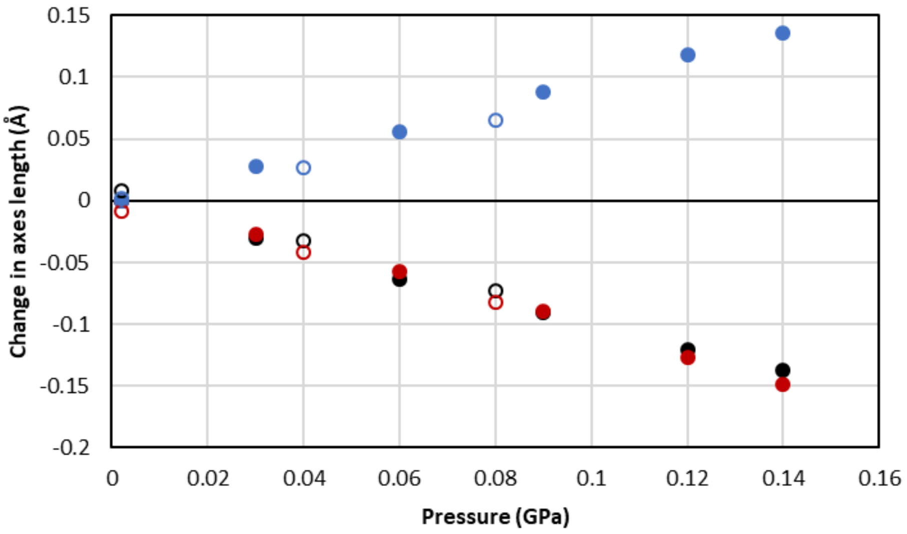

Figure S9. Changes in unit cell dimensions for **SHF-62-CHCl$_3$** under pressure of $CHCl_3$ in the SCC. Filled symbols correspond to measurements made upon increasing pressure; empty symbols correspond to decreasing pressure. *a*-axis is black, *b*-axis red, *c*-axis is blue.

S4.3 Fitting equations of state

For individual datasets, pressure as a function of unit cell volume was fitted to the third-order Birch-Murnaghan equation, as follows:

$$P(V) = \frac{3B_0}{2}\left[\left(\frac{V_0}{V}\right)^{\frac{7}{3}} - \left(\frac{V_0}{V}\right)^{\frac{5}{3}}\right]\left\{1 + \frac{3}{4}\,(B_0{}' - 4)\left[\left(\frac{V_0}{V}\right)^{\frac{2}{3}} - 1\right]\right\}$$

where, P is the pressure (GPa), V_0 is the reference volume at P=0; V is the deformed volume at pressure, P; B_0 is the bulk modulus and B_0' is the derivative of the bulk modulus with respect to pressure. B_0 and B_0' were obtained through the fitting of the equation to the experimental data.

Data were fitted using the PASCal software[S16,S17] and the fits are shown in Figure S10.

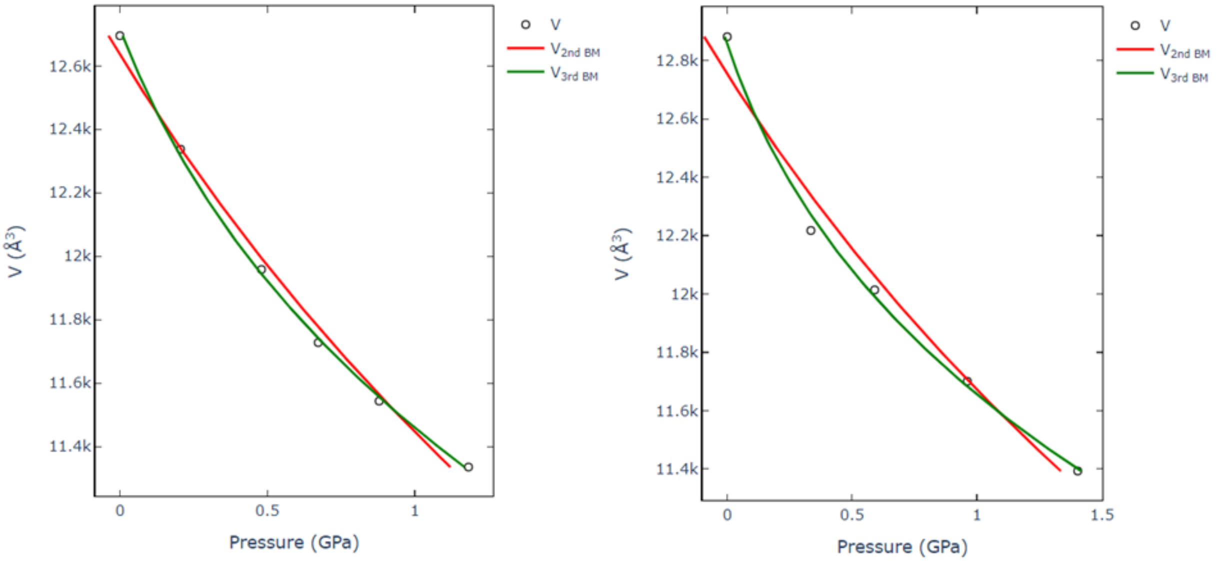

Figure S10. 2nd and 3rd order Birch-Murnaghan equations of state fits to the experimental data for (left) **SHF-62-DMF** and (right) **SHF-62-$CHCl_3$** in FC-70, obtained using the PASCal software.[S16,S17]

Table S6. Parameters for 3rd order Birch-Murnaghan equations of state fits.

	***B_0* (GPa)**	***V_0* (Å$_3$)**	***B_0'* (GPa)**	**Reference P (GPa)**
**SHF-62-DMF**	5.6(4)	12695.17(9)	12(2)	0
**SHF-62-$CHCl_3$**	4.9(9)	12860(2)	18(6)	0

S4.4 Structural analyses

S4.4.1 Carboxylate O···O vector hinges

We have previously established that the breathing motion of the MOF involves a hinge motion about the two carboxylate O···O vectors of the framework ligand.[S8,S9] The two independent hinge angles about the carboxyl O···O vectors associated with the framework ligand in **SHF-62** are shown in Figure S11.

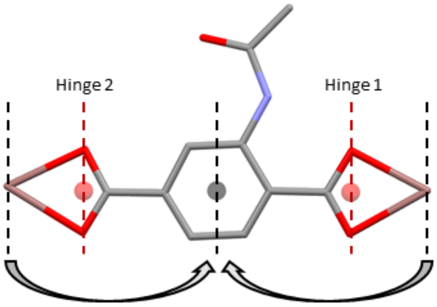

Figure S11. Schematic of hinges about the carboxylate O···O vectors for the framework ligand in SHF-62.

The average of the two hinge angles as a function of pore opening are presented in the manuscript (Figure 4b). In Figures S12 and S13 we show the individual hinge angle values as a function of pressure.

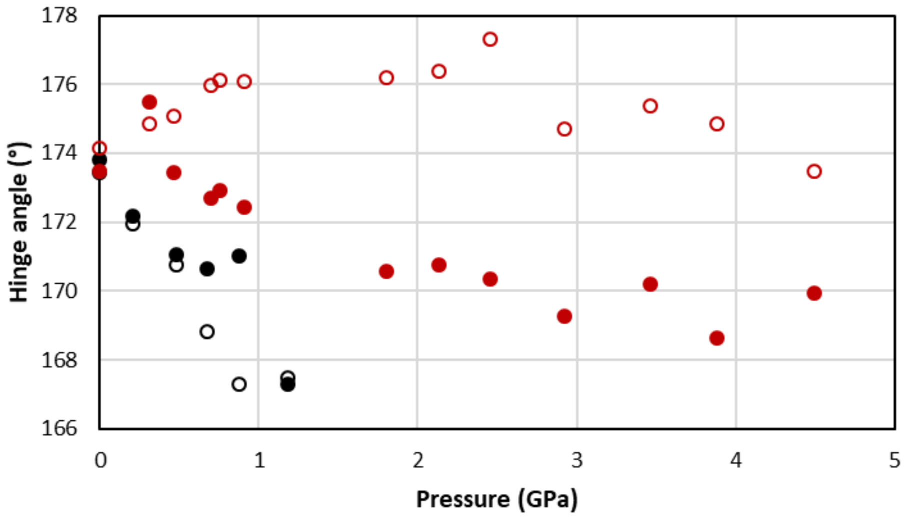

Figure S12. Hinge angles as a function of pressure presented for **SHF-62-DMF** in FC-70 (black) and DMF (red). Hinge 1 is represented by filled circles; hinge 2 by open circles.

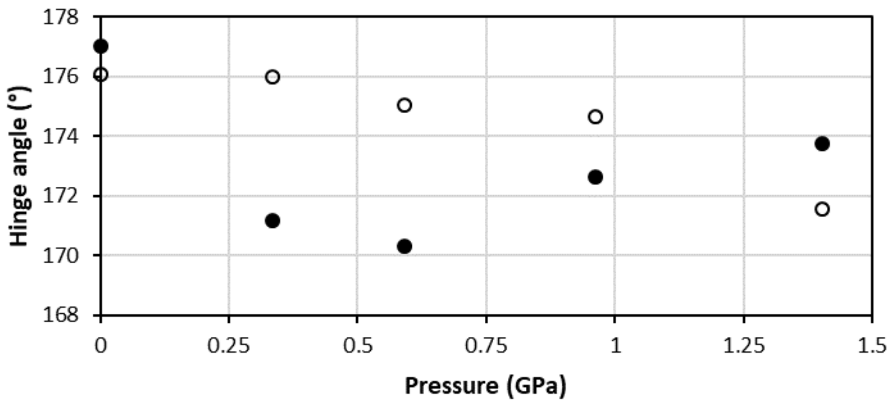

Figure S13. Hinge angles as a function of pressure presented for **SHF-62-CHCl$_3$** in FC-70. Hinge 1 is represented by filled circles; hinge 2 by open circles.

S4.4.2 Steric clash between pendant amide methyl groups

To explore the potential clash of pendant amide methyl groups from neighbouring ligands that gives rise to a ligand flip to alleviate steric clash during desolvation,[S9] we mapped the direction of motion and proximity of the neighbouring methyl groups as a function of pressure. In order to do so, we created artificial files containing atomic coordinates of only the methyl carbon atoms of the pertinent neighbouring groups. Their position within the unit cell could be visualized as a function of pressure by setting the unit cell origins in the same location and mapping each carbon atom by overlaying a series of these coordinates. Visualised below in Figure S14 are sets of the closest methyl pairs within the MOF pore space. In Figure S14a-c, a consistent view was chosen across the three mapped datasets to show the different ranges of methyl motion (with methyl···methyl distances at starting and final pressures). In Figure S14d, we visualise this motion within the MOF pore channel environment. Methyl···methyl distances are reported as the distance between the carbon atom of each methyl group.

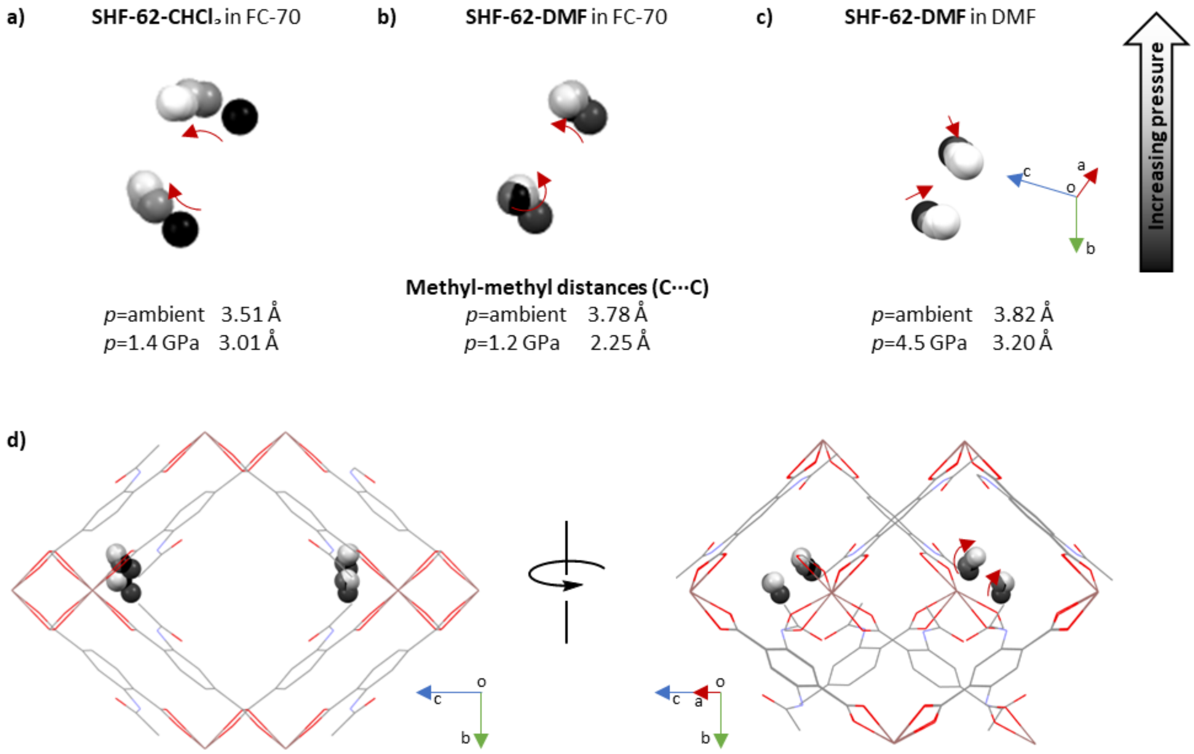

Figure S14. Methyl (carbon atom) motion upon increasing pressure in a) **SHF-62-CHCl$_3$** in FC-70, b) **SHF-62-DMF** in FC-70 and c) **SHF-62-DMF** in DMF (all DAC data), with methyl···methyl (C···C) distances indicated for *p*=ambient and p_{max}; d) methyl group motion modelled within the pore, for **SHF-62-DMF** in FC-70 using the ambient-pressure framework as a reference. Views shown are down the *a*-axis (left) and, for further clarity, down the [40−1] direction (right). The methyl carbon atom position at the initial pressure is shown in black, and as pressure increases the atom colour lightens. Arrows indicate direction of motion upon increasing pressure.

S5 References

S1. A. B. Pangborn, M. A. Giardello, R. H. Grubbs, R. K. Rosen F. J. Timmers, *Organometallics* **1996**, *15*, 1518–1520.

S2. C. J. McMonagle, D. R. Allan, M. R. Warren, K. V. Kamenev, G. F. Turner, S. A. Moggach, *J. Appl. Crystallogr.* **2020**, *53*, 1519–1523.

S3. H. Nowell, S. A. Barnett, K. E. Christensen, S. J. Teat, D. R. Allen, *J. Synch. Rad.* ***2012***, *19*, 435–441.

S4. CrysAlisPro. Single-crystal X-ray diffraction data collection and processing software, Rigaku Oxford Diffraction, Oxfordshire, UK, **2016**.

S5. G. Winter, *J. Appl. Cryst*. **2010**, *43*, 186-190.

S6. G. M. Sheldrick, *Acta Crystallogr.* **2008**, A*64*, 112–122.

S7. O. V. Dolomanov, L. J. Bourhis, R. J. Gildea, J. A. K. Howard, H. Puschmann, *J. Appl. Crystallogr.* **2009**, 42, 339-341.

S8. E. J. Carrington, C. A. McAnally, A. J. Fletcher, S. P. Thompson, M. Warren, L. Brammer, *Nat. Chem.* **2017**, 9, 882–889.

S9. E. J. Carrington, S. F. Dodsworth, S. van Meurs, M. R. Warren, L. Brammer, *Angew. Chem. Int. Ed*. **2021**, *60*, 17920–17924.

S10. I. D. H. Oswald, A. R. Lennie, C. R. Pulham, K. Shankland, CrystEngComm **2010**, *12*, 2533–2540.

S11. S. A. Moggach, D. R. Allan, S. Parsons, J. E. Warren, *J. Appl. Cryst.* **2008**, *41*, 249-251.

S12. G. S. Pawley, *J. Appl. Cryst.* **1981**, *14*, 357–361.

S13. TOPAS Academic Version 7, Coelho Software, 2020. *See http//www.topas-academic.net.*

S14. A. A. Coelho, J. Evans, I. Evans, A. Kern, S. Parsons, *Powder Diffr.* **2011**, *26*, S22–S25.

S15. A. A. Coelho, *J. Appl. Cryst.* **2018**, *51*, 210–218.

S16. M. J. Cliffe, A. L. Goodwin, *J. Appl. Crystallogr.* **2012**, *45*, 1321–1329.

S17. M. Lertkiattrakul, M. L. Evans, M. J. Cliffe, *J. Open Source Softw*. **2023**, *8*, 5556.
